# 

**Published:** 1893-07-15

**Authors:** 


CONTENTS.

English and American Doctors . . . . . . . . 241

Around the Hospitals . . . . . . . . . 242

Medical History from the Earliest Times. By E. T. Withington, M.A., M.B.Oxon. LXII.—The Surgery of the Eighteenth Century . . . . . . . . 243

Gleanings in Science and Medicine . . . . . . 244

The Relations of Hospitals to Public Health. By J. S. Billings, M.D. . . . . . . . . 245

Notes and News . . . . . . . . . . 248

The Hospital Clinic—Royal Infirmary, Edinburgh —On the Treatment of Pernicious Anæmia by Transfusion of Blood . 249

City of London Hospital for Diseases of the Chest, Victoria Park. Treatment of Pulmonary Phthisis—I. . 250

New Appliances and Things Medical.—Tabellæ—Messrs. Hockin, Wilson, and Co.'s Preparations—Milk of Magnesia—Levico Water—Rosbach Water . . . . . . . 251

Scraps and Gleanings . . . . . . . . . . 256

Annotations.—Can Doctors Joke?—Infants and Mailcarts—Poor Pay, Bad Work . . . . . . . . . . 247

New Drugs and Preparations.—Grindelia Robusta in Asthma and Chronic Bronchitis . . . . . . . . . 252

The Institutional Workshop—

Within the Hospitals.—Belle Vue Hospital, New York. By Our Special Correspondent . . . . . . . 253

Practical Departments.—Ward Waggons—Chamber Portable Closet . . . . . . . . . . . 254

Festival Dinners and Meetings.—National Dental Hospital—Poplar Hospital for Accidents—North London Hospital for Consumption—Society for the Study of Inebriety . . . 255

Construction Notes.—Additions to Cookridge Hospital . . 256

Editor's Letter Box.—Disinfection of Scarlet Fever by Antiseptic Inunction . . . . . . . . . . 256

The Book World of Medicine and Science . . . . vii

Medical Vacancies and Appointments . . . . . ix

EXTRA SUPPLEMENT.—THE NURSING MIRROR.

News from the Nursing World.—Nurses for Workhouses—Emergencies Intelligently Met—Incomplete—Pleasures Freely Provided—The Red Cross Flower Mission—Medals at Gravesend—A Capital Suggestion—District Nursing at Stalybridge—Another Nurses' Home—Queen's Nurses at Darlington—Worth Consideration—Medical Missions—Short Items—The Disabled Nurse . . . . . . . clii

Sanitation for Nurses. By A Sanitary Inspector. XIII.—Disinfectants (*continued*) . . . . . . . cliii

Educational Standards for Nurses. By Miss Isabel A. Hampton. I.—Hospital, District, and Private Nursing . cliv

Associations for the Supply of Cottage Nurses . . clv

Select Committee on Midwives . . . . . . clv

Appointments, clv; Death in Our Ranks . . . . clvi

Rabies and Glanders. I.—Rabies . . . . . clvi

Everybody's Opinion.—Lectures—Staffordshire Institution for Nurses . . . . . . . . . . clvi

A Novel Competition for Nurses—Notes and Queries clvii

For Reading to the Sick—Marriage.—London Hospital clv.i

The International Nursing Congress, Chicago. II.—Discussions on Nursing Topics . . . . . . . clviii

The Book World for Women and Nurses . . . . clix

